# Iron status in early infancy is associated with trajectories of cognitive development up to pre-school age in rural Gambia

**DOI:** 10.1371/journal.pgph.0002531

**Published:** 2023-11-01

**Authors:** Samantha McCann, Luke Mason, Bosiljka Milosavljevic, Ebrima Mbye, Ebou Touray, Alhassan Colley, William Johnson, Sarah Lloyd-Fox, Clare E. Elwell, Sophie E. Moore, the BRIGHT Study Team

**Affiliations:** 1 Department of Women and Children’s Health, King’s College London, London, United Kingdom; 2 Institute of Psychology, Psychiatry and Neuroscience, King’s College London, London, United Kingdom; 3 Department of Psychology, University of Cambridge, Cambridge, United Kingdom; 4 Medical Research Council Unit, The Gambia at the London School of Hygiene and Tropical Medicine, Fajara, The Gambia; 5 School of Sport, Exercise and Health Sciences, Loughborough University, Loughborough, United Kingdom; 6 Department of Medical Physics, University College London, London, United Kingdom; KEM Hospital Pune, INDIA

## Abstract

**Introduction:**

Iron deficiency is among the leading risk factors for poor cognitive development. However, interventions targeting iron deficiency have had mixed results on cognitive outcomes. This may be due to previous interventions focusing on the correction of iron deficiency anaemia in late infancy and early childhood, at which point long lasting neural impacts may already be established. We hypothesise that the relationship between iron status and cognitive development will be observable in the first months of life and will not be recovered by 5 years of age.

**Methods:**

Using data from the Brain Imaging for Global Health (BRIGHT) Study in Gambia (n = 179), we conducted mixed effects modelling to assess the relationship between iron status at 5 months of age and trajectories of cognitive development from 5 months– 5 years using (i) a standardised measure of cognitive development (Mullen Scales of Early Learning) and (ii) an eye-tracking assessment of attention processing (visual disengagement time).

**Results:**

All infants were iron sufficient at 1 month of age. At 5 and 12 months of age 30% and 55% of infants were iron deficient respectively. In fully adjusted analyses, infants in the lowest tercile of soluble transferrin receptor (sTfR) (best iron status) achieved MSEL Cognitive Scores on average 1.9 points higher than infants in the highest sTfR tercile (p = 0.009, effect size = 0.48). There was no evidence that this group difference was recovered by 5 years of age. Infants in the lowest sTfR tercile had visual disengagement time 57ms faster than the highest tercile (p = 0.001, effect size = 0.59). However, this difference diminished by early childhood (p = 0.024).

**Conclusion:**

Infants are at risk of iron deficiency in early infancy. A relationship between iron status and cognitive development is apparent from 5 months of age and remains observable at 5 years of age. One mechanism by which iron availability in early infancy impacts brain development may be through effects on early attentional processing, which is rapidly developing and has substantial nutritional requirements during this period. To support neurocognitive development, prevention of iron deficiency in pre- and early postnatal life may be more effective than correcting iron deficiency once already established.

## Introduction

Iron deficiency (ID) is the most common micronutrient deficiency worldwide, and is particularly prevalent among pregnant women, infants and young children due to high iron demands during periods of rapid growth [1]. Iron deficiency anaemia (IDA) has been reported as one of the major factors preventing 250 million children under five years of age from realising their developmental potential [2]. There is strong mechanistic evidence suggesting that this link between IDA and early child development is due to the important roles of iron in neurodevelopmental processes including myelination, hippocampal development, and dopaminergic neurotransmission [3]. However, the results of interventions attempting to support neurocognitive development through iron supplementation have been disappointing [4–8], and currently there is no consensus on the threshold of impact or optimal timing or composition of intervention. A recent systematic review reported that this may be due to a mismatch between the mechanistic evidence, which focuses on the importance of preventing ID in prenatal and early postnatal development [9,10], and that of studies and trials in humans, which focus largely on the correction of IDA in late infancy and early childhood [11]. This may be due to an assumption that term infants receive adequate iron endowment in utero to maintain iron status for the first 6 months of life and are therefore protected from iron deficiency during this period [12]. As well as highlighting the neglect of the first 6 months of life, the review concluded that there was insufficient research conducted in populations at highest risk of ID (particularly sub-Saharan Africa) and that statistical analyses often failed to adjust for confounding effects, such as socio-economic status or gestational age at birth. These weaknesses limit interpretation and put the results at high risk of bias. Finally, the review concluded that the tools used to measure neurocognitive development were mostly confined to behavioural assessment which can be subjective, restrict mechanistic insights and may lack sensitivity in infancy, when the behavioural repertoire is limited [11].

The aim of this study was to address these research gaps by investigating the relationship between iron status in early infancy (5 months of age) and longitudinal trajectories of neurocognitive development across infancy and early childhood, using data from the Gambian cohort of the Brain Imaging for Global HealTh (BRIGHT) study and BRIGHT Kids Study (https://www.globalfnirs.org) This cohort of 200 mother infants dyads was recruited from the rural West Kiang region of the Gambia,, where ID and IDA are widespread among infants [13].

Flexibility in visual attention develops around 3–6 months of age and is foundational to learning [14]. Rapid development requires additional nutritional resources, and therefore may be particularly sensitive to nutritional deficiencies, within this period [15]. Visual attention, as a reflection of processing speed, is also mechanistically linked to the iron-dependent process of myelination [16].

As well as mechanistic links to iron status, the use of eye tracking has the added benefits of being sensitive and objective [17] and not relying on social interaction from participating infants, preventing the confound of cognitive abilities with temperament. In addition, looking behaviours have previously been shown to be predictive of later developmental outcomes [15] suggesting that measurable differences are meaningful for development in the longer term.

We hypothesised that iron status at 5 months of age would be associated with neurocognitive outcomes with lasting effect, and that the objective measure of attentional flexibility (visual disengagement time) would be more sensitive to early iron status than the global measure of cognitive development (Mullen Scales of Early Learning (MSEL) Cognitive Score).

## Methods

The data presented was drawn from the Brain Imaging for Global Health (BRIGHT) study, which was conducted in the West Kiang Region of The Gambia from July 2015 to July 2020, and the BRIGHT Kids study conducted between July 2021 and March 2022. BRIGHT was a longitudinal cohort study conducted collaboratively by Medical Research Council Unit The Gambia at the London School of hygiene and Tropical Medicine, University College London, University of Cambridge, Birkbeck University of London, King’s College London and Cambridge University Hospitals. The aim of the BRIGHT study was to investigate trajectories of neurocognitive [18] development and associated social, environmental, and nutritional factors across infancy and early childhood. Women were recruited in pregnancy and then followed up along with their new infant for 2 years postnatally. BRIGHT-Kids was an additional cross-sectional follow-up study of the Gambian BRIGHT cohort at pre-school age (3–5 years). Measures of infant neurocognitive development were undertaken at 5, 8, 12, 18 and 24 months of age as well as at 3–5 years. A range of neurocognitive measures were implemented within BRIGHT, two of which are reported on within the presented study and described in more detail below. Infant iron status was determined through analysis of blood samples collected at 1, 5, 8 and 12 months of age.

Ethical approval was given by the joint Gambia Government/Medical Research Council Unit The Gambia at the London School of Hygiene and Tropical Medicine Ethics Committee, and informed consent was obtained in writing, or via thumbprint if individuals were unable to write, from participating pregnant women.

### Iron and inflammatory status

Prevalence of iron deficiency was assessed at 1, 5 8 and 12 months of age. Iron status at 5 months of age was investigated in relation to trajectories of cognitive development. There is no consensus as to which biomarker of iron status is most closely related to cognitive development, nor is there an established threshold of impact. Therefore, we investigated iron deficiency versus iron sufficiency and three individual biomarkers (i) ferritin concentration (ii) soluble transferrin receptor (sTfR) concentration and (iii) haemoglobin (Hb) concentration, each of which were adjusted for c-reactive protein (CRP) in line with recommendations from the Biomarkers Reflecting Inflammation and Nutritional Determinants of Anaemia (BRINDA) project [19].

Capillary (1 & 8 months of age) or venous (5 & 12 months of age) blood samples were collected at infant study visits and Hb was measured immediately using a Medonic-M32 analyser (Boule Diagnostics AB, Stockholm, Sweden). Plasma was then separated and stored at -70°C on the day of collection. Following completion of the 12 month study visits, plasma samples were thawed and assays of ferritin, sTfR and CRP, were completed using the COBAS INTRGRA 400 (Roche Diagnostic Corp., Indianapolis, USA).

### Mullen scales of early learning

The MSEL is an assessment which measures cognitive and motor development through the direct observation of behaviour. It has previously been adapted by our team for use in this population in The Gambia [20]. The MSEL is comprised of a gross motor scale and four cognitive scales: visual reception, fine motor, receptive language, expressive language. The sum of the raw scores in each of the cognitive scales gives the Cognitive Score, which was used as the outcome from this assessment within the following analyses. The age-standardised Early Learning Composite Score (ELC), which is commonly used as the outcome measure of this assessment [21], was not used in this study for two reasons. Firstly, ELC scores are standardised based on US norms which may not be appropriate for this cohort. Secondly, raw scores were better suited to the analytic plan, which aimed to map the trajectory of development.

The MSEL assessment was conducted by fully trained members of the BRIGHT field team in the Mandinka language, the native language of the mothers and infants in the study.

### Visual disengagement time

The second neurodevelopmental outcome reported in this study was an assessment of the flexible switching of visual attention, known as disengagement, measured using optical eye tracking technology. Visual disengagement time was derived from infant responses to a gap-overlap paradigm, based on a task developed by Elsabbagh et al. [22,23] and was one component of the larger eye tracking battery implemented within the BRIGHT study. Details of the equipment and assessment procedure are reported in the Supplementary Methods. In summary, the paradigm assessed the difference in infant saccadic reaction time when switching their visual attention from existing visual stimulus in the centre of the screen to a new visual stimulus at the periphery of the screen, in the presence or absence of the centrally located stimulus. Disengagement time decreases with age [24], therefore faster disengagement represented a more mature response.

### Data analysis

Infants were included in analyses if they had measures of iron status and inflammation (ferritin, c-reactive protein (CRP), sTfR, Hb) at 5 months of age, and cognitive measures (MSEL and disengagement time) for at least one time point between 5 months and 3–5 years of age (*n* = 179).

For samples obtained at 5, 8 & 12 months of age, ferritin was log transformed to ensure a normal distribution. CRP was log transformed at all time points for the same reason. sTfR, Hb and all outcome variables were normally distributed, so transformation was not necessary. When describing the iron status of the cohort, iron deficiency was defined as ferritin < 12μg/L or <30 μg/L if CRP>5mg/L at all time points. Tercile groups were established for Hb, ferritin and sTfR so that each infant was determined to have ‘high’, ‘medium’ or ‘low’ concentration of each biomarker.

Individual trajectories of developmental performance (MSEL cognitive score and visual disengagement time) from 5 months to 5 years of age were constructed using mixed effects modelling with repeated outcome measures clustered within infants. The models included a random intercept and random slope on age, centred at 5 months of age, meaning that the model intercept could be interpreted as developmental performance at 5 months of age. The fractional polynomial command in Stata 17 (StataCorp 2021, College Station, Texas) was used to identify the best fitting set of polynomial age terms to fit each model.

Once the basic models of MSEL Cognitive Score and disengagement time trajectories were finalised, each iron biomarker was added to a separate model as fixed effects using the tercile groups. Model fit was then reassessed to determine whether it was improved when compared to the basic model. For all biomarkers, the tercile representing poorest iron status was used as the base group and therefore not entered into the model. For Hb and ferritin this corresponded to the lowest tercile, whereas for sTfR this corresponded to the highest tercile. To investigate whether the developmental impact of early iron status was maintained over time, age*iron interaction terms were included in the models. Finally, models were adjusted for variables which were determined a priori as factors mechanistically related to the exposure (iron status) and the outcome (cognitive development) but not on the causal pathway. The chosen variables were; infant sex, season of infant birth, maternal education, infant gestational age at birth, infant length and CRP at 5 months of age. Further details on the collection of these variables and the rationale for choosing each is outlined in the S1 Text.

At every stage of model development, model fit was assessed using the likelihood ratio statistic and Bayesian Information Criterion (BIC). Diagnostic plots were generated to check residuals for normality and homogeneity (S1–S6 Figs). An unstructured intercept-slope covariance matrix was specified in all models. Observations with very large residuals were removed from the analysis under the assumption of measurement error (MSEL n = 30, visual disengagement time n = 30). Final model fit was assessed using the residual standard deviation (RSD). The RSD for the cognitive score trajectory was 4.2 points and for disengagement time was 55ms which are within normal measurement error for the respective assessments and therefore indicate adequate model fit.

## Results

**Fig 1:**
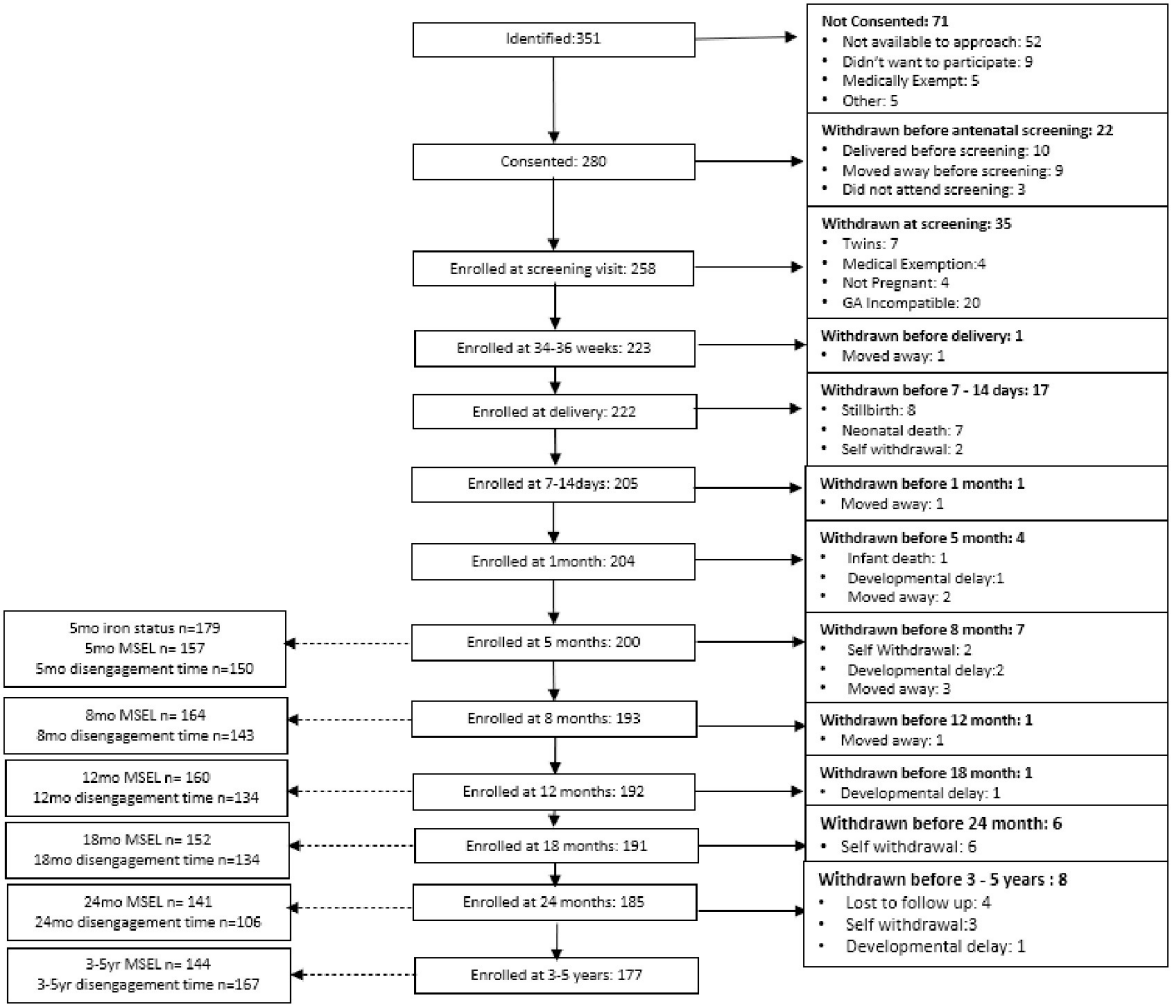
Flowchart of participant retention and data collection. GA incompatible: gestational age of participant incompatible with study recruitment schedule. The study was set up so that a maximum of 15 expected deliveries were accepted each month, in order to ensure the study team could fulfil the requirements of the study. Data of withdrawn participants was retained in analysis with the exception of those diagnosed with a developmental delay. In these cases, all participant data was excluded from the dataset.

A total of 351 pregnant women were identified as eligible for inclusion in the study, of which 280 consented to join and 222 remained within the study at the time of delivery (Fig 1). Eight infants were stillborn, and a further 45 were lost to follow up between birth and 3–5 years of age, representing 20.3% of live births. Complete iron and inflammatory data were available for 98.1% of infants who attended the study visit at 5 months of age. Over 70% of attending infants completed the developmental outcome measures at all time points, except for visual disengagement time at 24 months. Lower completion rates at 24 months of age were due to interruption caused by the Covid 19 pandemic (S1 and S2 Tables).

### Participant characteristics

Within this study sample the mean age of mothers was 30 years, and most mothers were multiparous (88.8%). Parental education was very low, with 59% of mothers and 55% fathers having no formal education. Socio-economic status of the sample as a whole was also, very low, with few participants having access to piped water (1.3%) or electricity (0.7%) within their family compound. The most reported occupation for both mothers and fathers was farming.

In terms of infant nutrition, the median duration of exclusive breastfeeding was 5.8 months, and 44.2% infants were exclusively breastfed to 6 months of age (Table 1). There was some evidence that infants who were exclusively breastfed to 5 months of age or beyond were more likely to be iron deficient at this age than those who were not exclusively breastfed (S7 Fig). Mean infant LAZ and WAZ declined from birth to 12 months of age, as did mean ferritin and haemoglobin concentration. sTfR increased across the same period, reflecting that sTfR is an indicator of iron sparsity as opposed to iron availability (Table 2).

**Table 1:** Participant characteristics: Demographics.

	N	Mean (SD)/ Percentage/ Median (IQR)
***Family***		
Maternal age (years)	178	29.9 (6.6)
Maternal anaemia in late pregnancy (%)^1^	150	56.7
Primiparous (%)^2^	178	11.2
Polygamous marriages (%)	152	40.8
Maternal education^3^ (years)	152	0 (0–5)
Paternal education^3^ (years)	151	0 (0–12)
Farming Mothers (%)	151	68.9
Farming Fathers (%)	152	44.1
Piped Water in family compound (%)	152	1.3
Electricity in family compound (%)	152	0.7
***Infant***		
Male (%)^2^	179	49.7
Gestational age at birth (weeks)	179	39.8 (1.9)
Duration of exclusive breastfeeding (months)^3^	172	5.8 (5.0–6.8)
Exclusively breastfed to 6 months^2^	172	44.2

Results presented as mean (SD) unless stated otherwise.

^2^Presented as percentage.

^3^ Presented as median and IQR.

^4^Hungry Season = June to October.

Bold font indicates p<0.05.

**Table 2:** Participant characteristics: Nutritional markers.

	Mean (SD)/ Median (IQR)/ Percentage				
	Birth	1 month	5 months	8 months	12 months
WAZ	-0.59 (0.74)	-0.51 (0.93)	-0.62 (0.99)	-0.82 (0.96)	-0.97 (0.95)
LAZ	-0.35 (0.89)	-0.84 (0.96)	-0.58 (0.98)	-0.82 (0.96)	-1.05 (1.00)
WLZ	-0.58 (1.20)	0.35 (1.10)	-0.27 (1.04)	-0.43 (1.01)	-0.62 (1.00)
Ferritin (μg/L)	-	276.45 (215.80–368.10)	36.00 (16.00–59.40)	22.40 (11.50–39.20)	15.95 (8.10–30.80)
Hb (g/dL)	-	12.15 (1.91)	10.78 (0.90)	10.15 (1.20)	10.27 (0.96)
sTfR (mg/L)	-	2.18 (0.71)	4.26 (1.60)	5.36 (1.38)	6.12 (1.68)
Anaemic (%)	-	28.24	53.59	77.19	77.91
Iron Deficient (%)		0	29.59	38.41	55.33
IDA (%)	-	0	19.08	29.53	47.13

SD; Standard Deviation, IQR; Interquartile Range, Hb; haemoglobin, WAZ; weight-for-age-z score, LAZ; length-for-age z score, WLZ; weight-for-length z score, sTfR; soluble transferring receptor. (WAZ/ LAZ/WLZ 1month n = 180, 5month n = 193, 8month n = 186, 12month n = 186. Ferritin/ Hb/sTfR/ Iron deficient 1month n = 170, 5month n = 181, 8month n = 171, 12month n = 172.

Iron/anaemia markers not measured at birth.

Anaemic defined as haemoglobin <11g/dL.

Iron deficient defined as ferritin <12μg/L if CRP < 5mg/L or ferritin <30μg/L if CRP≥ 5mg/L, IS; iron sufficient defined as ferritin ≥12μg/L if c reactive protein (CRP) < 5mg/L or ferritin ≥ 30μg/L if CRP ≥ 5mg/L.

IDA; iron deficiency anaemia defined as concurrently iron deficient (as ferritin <12μg/L if CRP < 5mg/L or ferritin <30μg/L if CRP≥ 5mg/L, IS; iron sufficient defined as ferritin ≥12μg/L if c reactive protein (CRP) < 5mg/L or ferritin ≥ 30μg/L if CRP ≥ 5mg/L) and anaemic (haemoglobin <11g/dL).

### Iron status across infancy

**Fig 2:**
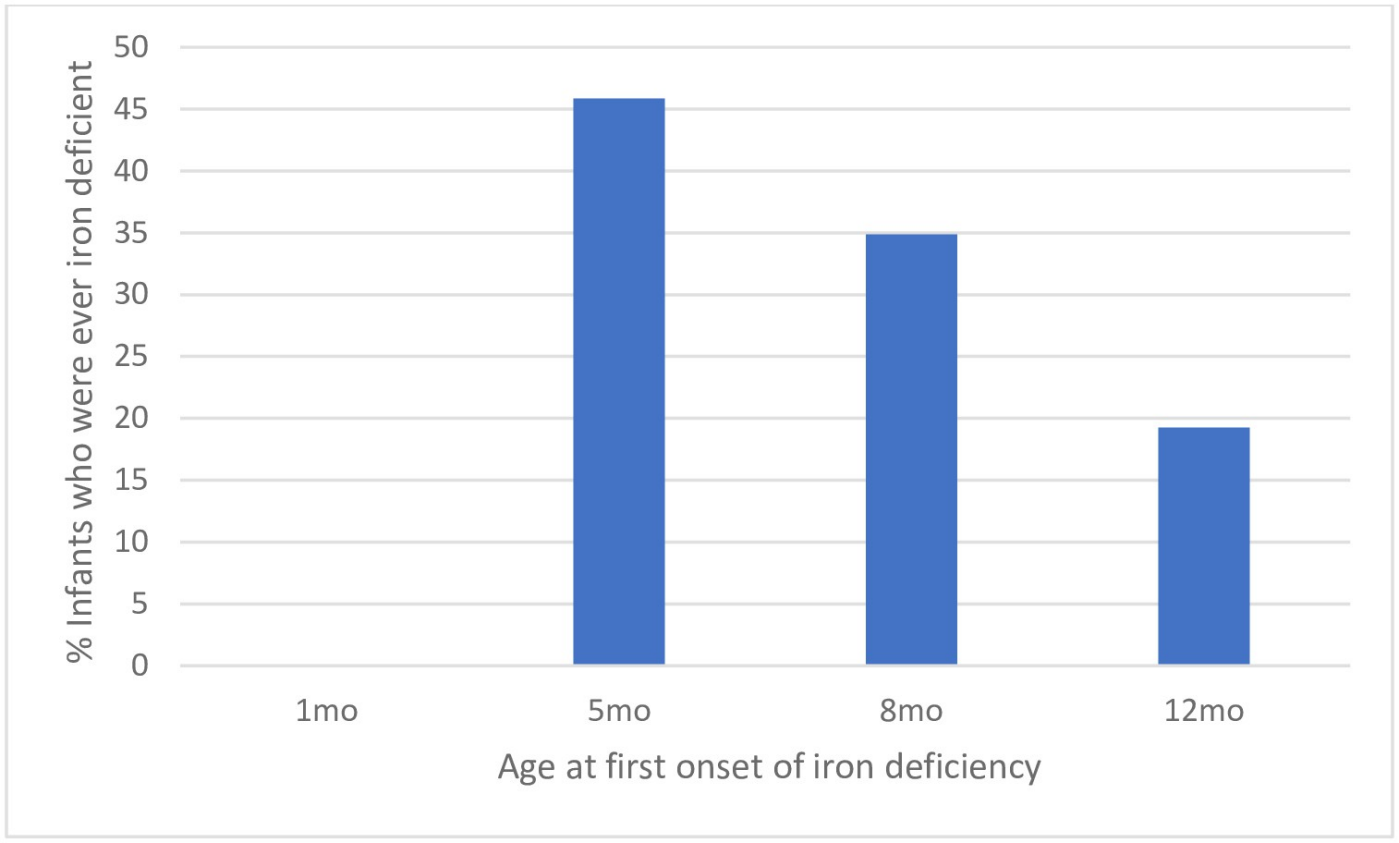
Incidence of iron deficiency onset. N = 109 (Infants met the criteria for iron deficiency at 1, 5, 8 or 12 months) iron deficiency defined as ferritin <12μg/L if CRP < 5mg/L or ferritin <30μg/L if CRP≥ 5mg/L.

All infants were iron sufficient at 1 month of age. However, the prevalence of iron deficiency increased from 29.6% at 5 months of age to 55.3% at 12 months of age. To investigate the time of iron deficiency onset, we categorised infants by time point (1, 5, 8 or 12 months) at which they first became iron deficient. Although the overall prevalence of iron deficiency was highest at 12months of age, the highest incidence of iron deficiency onset was between 1–5 months of age. Infant who became iron deficient between 1–5 months of age accounted for 46% of all iron deficient infants in the study (Fig 2).

### Neurocognitive trajectories: MSEL cognitive score from 5months- 5 years of age

In unadjusted analyses, for infants who were iron sufficient versus iron deficient (based on ferritin) at 5 months of age, no difference was identified in the cross-sectional relationship or longitudinal trajectory of MSEL Cognitive scores from 5 months and 5 years of age. Similar results were observed when comparing infants who had iron deficiency anaemia (IDA) at 5 months of age and those who did not.

**Fig 3:**
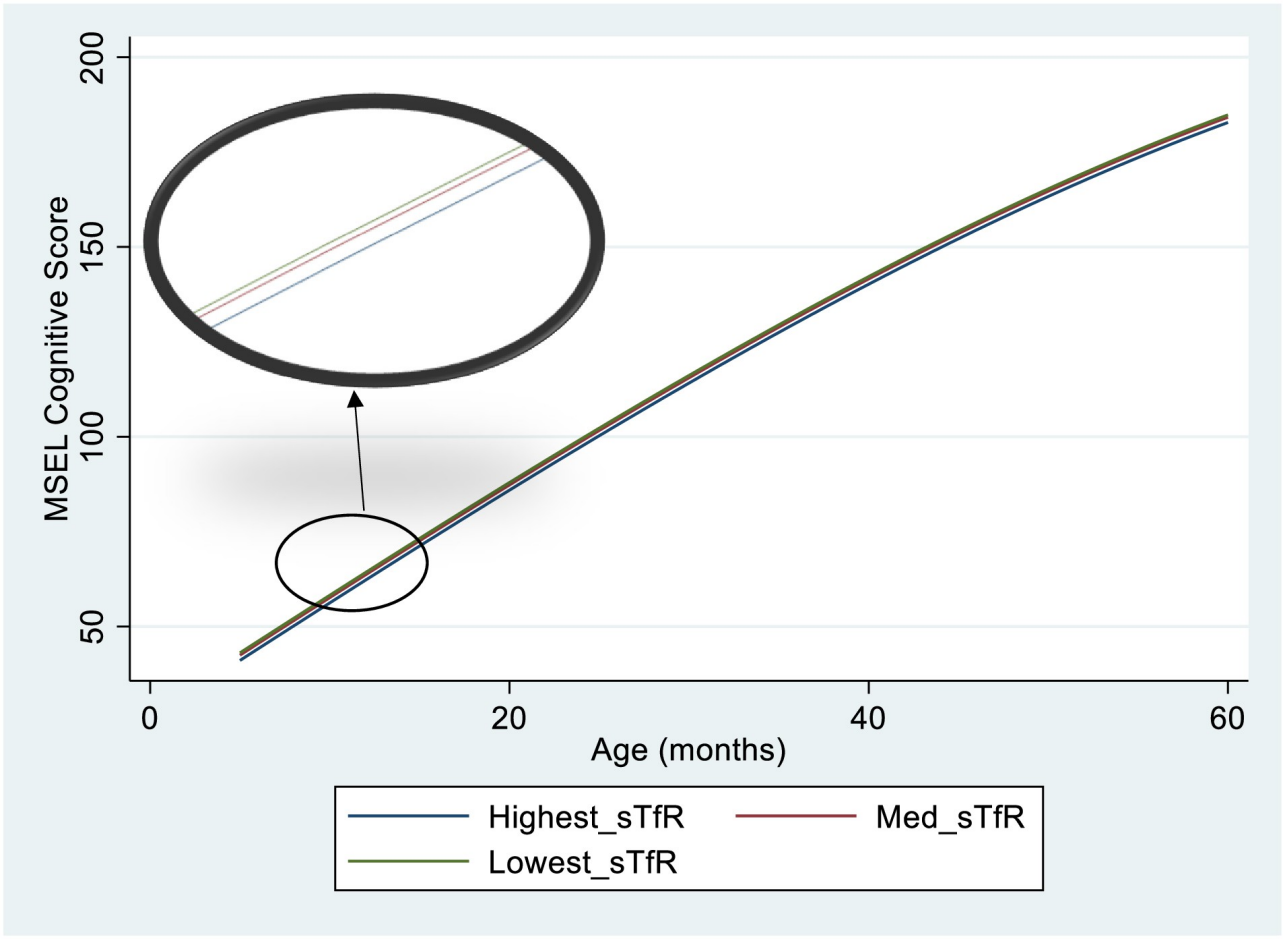
MSEL cognitive score trajectories by sTfR tercile at 5 months of age. The Figure shows the modelled mean trajectories of MSEL Cognitive score from 5 months to 5 years of age, for each tercile of sTfR at 5months of age. The large oval shows a magnified version of the section of the graph highlighted in the small oval. Infants in the lowest tercile of sTfR scored on average 2 points higher than those on the highest tercile at 5 months of age. This group difference was still observable at 5 years of age. Model Specification; MSEL Cognitive score = Bl*Age + B2*Age^3^ + B3*Med*sTfR + B4*Lowest_sTfR + Constant Age interactions were not included in the equation as they did not significantly improve the fit of the model.

Compared to infants in the high tercile for sTfR (poorest iron status) infants in the medium and low sTfR terciles achieved MSEL Cognitive Scores 1.4 and 2.0 points higher, respectively at 5 months of age, corresponding to a small- medium effect size (Low sTfR; p = 0.003 effect size = 0.51, Medium sTfR; p = 0.031, effect size = 0.35). In adjusted analysis, group differences were only observed between the high sTfR and low sTfR groups (Table 3). The inclusion of age interaction terms did not improve the model fit, suggesting that group differences in MSEL Cognitive Score by sTfR Tercile were stable across the assessed period (5 months—5 years of age). Fig 3 shows the trajectories of MSEL Cognitive Scores for each Tercile of sTfR.

**Table 3:** Iron biomarkers and trajectories of MSEL cognitive score.

	Unadjusted*				Effect Size	Adjusted**				Effect Size
	Coeff	P value	Age interaction	P value		Coeff	P value	Age interaction	P value	
**Ferritin**										
Low base group)										
Med	0.08	0.902	-0.05	0.532	0.02	0.44	0.540	-0.08	0.326	0.11
High	0.25	0.709	-0.02	0.793	0.06	-0.37	0.594	0.04	0.621	0.10
**Hb**										
Low (base group)										
Med	-0.92	0.157	0.15	0.034	0.23	-0.95	0.176	0.19	0.017	0.24
High	0.48	0.481	0.10	0.145	0.12	0.49	0.502	0.07	0.377	0.12
**sTfR**										
High (base group)										
Med	1.39	**0.031**	-0.09	0.198	0.35	1.28	0.069	-0.02	0.779	0.33
Low	2.00	**0.003**	-0.03	0.658	0.51	1.89	**0.009**	-0.01	0.982	0.48

Hb; haemoglobin.

sTfR; soluble transferrin receptor.

Low/ Med/ High represent terciles of each biomarker; low = lowest tercile, med = middle tercile, high = highest tercile. Higher haemoglobin and higher ferritin both indicate better status whereas higher sTfR indicates poorer status. For each biomarker the tercile representing poorest status was used as the base group (Ferritin base = Low, Hb base = low, sTfR base = high). The co-efficients in each row of the table therefore represent the comparison between the base group and the group named in the first column.

*Unadjusted models included age terms (age & age^3^), biomarker terciles, biomarker-age interaction terms for Terciles included in the model and log c-reactive protein. Observations included = 837, Infants included = 177, mean observations per infant = 4.7.

** (S3–S5 Tables). In addition to the variables included in the unadjusted models, adjusted models included sex, Fourier terms for seasonality, infant height, infant gestational age at birth and maternal education. Full details are available in the Supplementary Methods. Observations included = 430, infants included = 110, mean observation per infant = 3.9.

Effect Size = (group difference/ Standard deviation of base group).

No differences in trajectory of MSEL cognitive score were identified when assessed by Tercile of Hb or Ferritin.

### Neurocognitive trajectories: Visual disengagement time from 5 months—5 years of age

In line with the MSEL results, no difference in trajectory of visual disengagement time was observed between infants who were iron deficient or IDA and those who were not at 5 months of age. Neither were differences observed when investigating trajectories by Tercile groups of ferritin at 5 months of age (Table 4).

**Table 4:** Iron biomarkers and trajectories of visual disengagement time.

	Unadjusted*				Effect Size 5 mo	Adjusted**				Effect Size 5mo
	Coeff	P value	Age interaction	P value		Coeff	P value	Age interaction	P value	
**Ferritin**										
Low (Base Group)										
Med	-13.99	0.297	0.11	0.770	0.15	-27.95	0.119	2.65	0.023	0.30
High	-17.09	0.204	0.30	0.432	0.18	-25.93	0.145	2.42	0.032	0.28
**Hb**										
Low (Base Group)										
Med	-6.24	0.630	0.21	0.566	0.07	-3.85	0.824	0.06	0.957	0.05
High	-32.93	**0.014**	0.81	**0.037**	0.38	-28.32	0.115	0.58	0.612	0.33
**sTfR**										
High (Base Group)										
Med	-26.03	**0.048**	0.35	0.357	0.27	-29.49	0.093	1.19	0.301	0.31
Low	-46.53	**<0.001**	0.86	**0.028**	0.49	-56.61	**0.001**	2.58	**0.024**	0.59

Hb; haemoglobin.

sTfR; soluble transferrin receptor.

Low/ Med/ High represent terciles of each biomarker; low = lowest tercile, med = middle tercile, high = highest tercile. Higher haemoglobin and higher ferritin both indicate better status whereas higher sTfR indicates poorer status. For each biomarker the tercile representing poorest status was used as the base group (Ferritin base = Low, Hb base = low, sTfR base = high). The co-efficients in each row of the table therefore represent the comparison between the base group and the group named in the first column.

*Unadjusted models included age terms (age, ln(age), (ln(age)^)2^), biomarker terciles, biomarker-age interaction terms for Terciles included in the model and log C- reactive protein. Observations included = 721, Infants included = 180, mean observations per infant = 4.0 (S6–S8 Tables).

** In addition to the variables included in the unadjusted models, adjusted models included sex, Fourier terms for seasonality, infant height, infant gestational age at birth and maternal education. Full details are available in the Supplementary Methods. Observations included = 481, infants included = 152, mean observation per infant = 3.2.

Effect Size = group difference/ Standard deviation of base group.

However, in unadjusted models infants in the high Hb tercile at 5months (best haemoglobin status) had visual disengagement time on average 33ms faster than those in the low Hb tercile (p = 0.014). Although, there was evidence that this group difference diminished as age increased (age interaction; p = 0.037) with infants in all groups performing similarly by pre-school age. Results for sTfR followed a similar pattern with infants in the low sTfR tercile of (best iron status) having visual disengagement time on average 47ms faster than those in the high sTfR tercile (worst iron status) at 5 months of age, with group differences diminishing with increasing age (age interaction p = 0.028) (Table 4).

**Fig 4:**
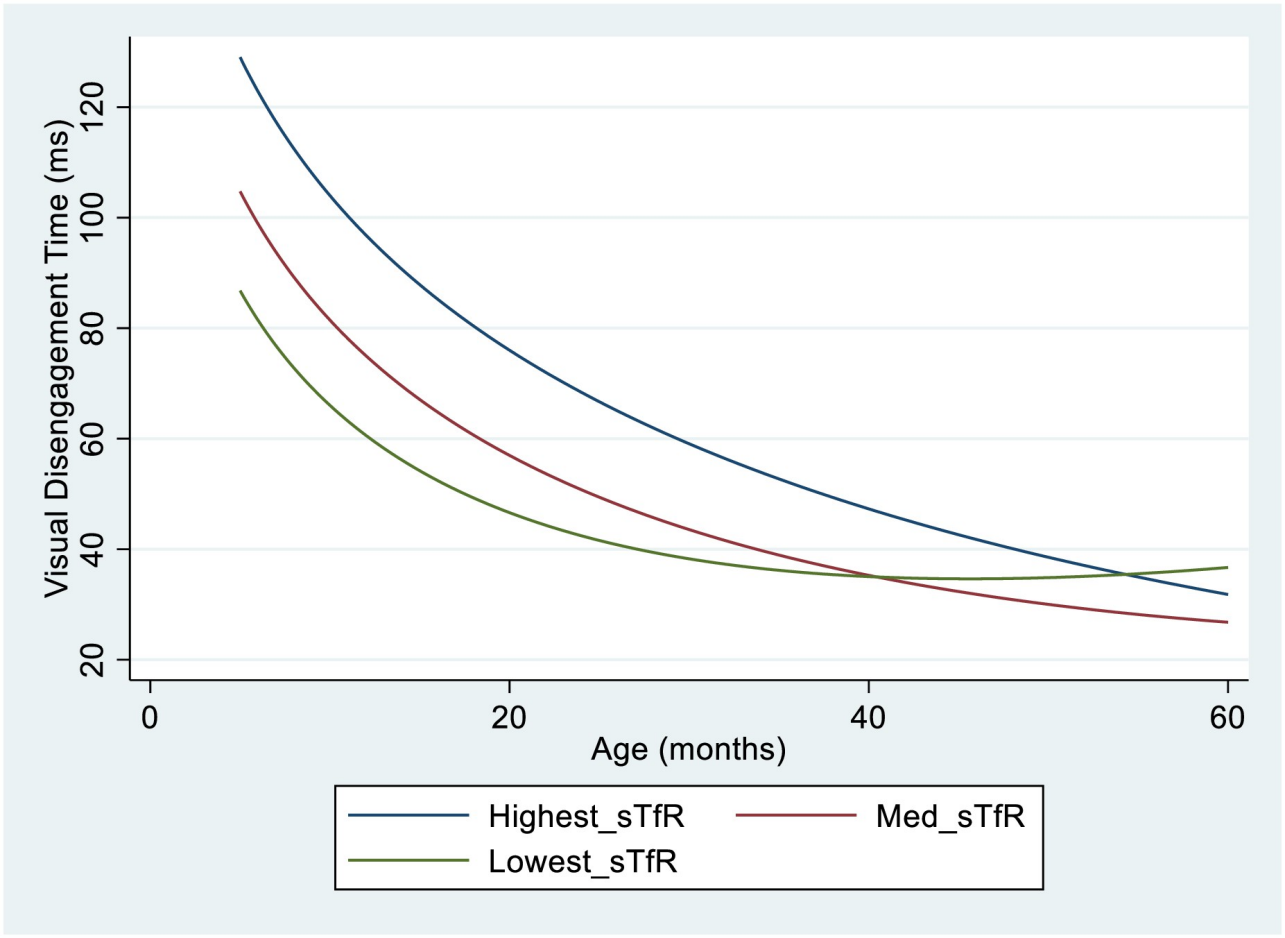
Visual disengagement time trajectories by sTfR tercile at 5months of age. The Figure shows the modelled mean trajectories of Visual Disengagement Time from 5 months to 5 years of age, for each tercile of sTfR at 5months of age. Infants in the lowest tercile of sTfR responded on average 47ms faster than those in the highest tercile of sTfR at 5 months of age. However, this group difference diminished from around 20 months of age, with negligible group differences by 40 years of age. Model Specification; Visual Disengagement time = Bl*Age + B2*ln(Age) + B3*(ln(Age)^2^ + B4*Med*sTfR + B5*Lowest_sTfR B6*Med_sTfR_Age + B7*Lowest sTfR_Age + Constant.

In adjusted analyses, the only group difference to remain significant was that between the highest and lowest terciles of sTfR. The trajectories of visual disengagement time by sTfR tercile are displayed in Fig 4.

## Discussion

We present data supporting previous findings that infants born in this region of rural sub-Saharan Africa are vulnerable to iron deficiency within the first six months of life [13], even when exclusively breastfed in accordance with WHO Infant and Young Child Feeding recommendations [25]. Further, infants with the worst functional iron status (measured by sTfR) at 5 months of age had poorer neurocognitive outcomes at that age, and this pattern tracked to 5 years of age, suggesting a long-lasting impact of poor iron availability in early infancy. These data highlight the potential for early iron interventions to support infant neurodevelopment in populations with a high burden of maternal anaemia. These data may also suggest that sTfR may be a more useful indicator than the widely used definition of iron deficiency (using ferritin and CRP) for identifying infants at risk of neurocognitive consequences of insufficient iron.

The increase in prevalence of iron deficiency in the first year of life that was observed in this study mimics findings from across sub-Saharan Africa. A recent publication including almost 5000 infants and children from five African countries similarly showed that iron deficiency increases across infancy, peaks at 1 year of age and then decreases thereafter [26]. However, few other studies have investigated the timing of iron deficiency onset and many studies exclude infants under 6 months of age from analyses of iron deficiency and anaemia [27–29]. Therefore, it is not certain whether the observed pattern of greatest iron deficiency incidence between 1–5 months of age is similar across the sub-region. We identified some evidence that infants who were still exclusively breastfed at 5 months of age were more likely to be iron deficient. However, this contrasts with findings from a cohort in rural Kenya (n = 134), which showed that infants who were exclusively breastfed for 6 months or longer had better iron status at 6–10 months of age [30]. That being said, the prevalence of iron deficiency and anaemia was very high (over 50% for all measures) in both groups and most of the ‘non-exclusively breastfed’ group were exclusively breastfed for at least 4 months [30]. The many benefits of exclusive breastfeeding to 6 months of age are well characterised and cannot be ignored [31]. However, it is also well known that breastmilk contains little iron. Therefore, prolonged breastfeeding can be detrimental to infant iron status if hepatic iron stores built up in-utero are inadequate as a result of factors such as maternal anaemia or pre-term birth [31].

Our study has shown that inadequate circulating iron (based on sTfR) under 6 months of age may have long term consequences for the developing brain. Therefore, further intervention in pregnancy or early infancy may be needed, alongside exclusive breastfeeding, to ensure infants receive all the benefits of exclusive breastfeeding and avoid nutritional deficiencies. Other than delayed cord clamping, there are currently no interventions widely recommended to support infant iron status in the first 6 months of life.

It is interesting that cognitive performance was consistently associated with sTfR in this analysis, but not ferritin. Ferritin is a measure of iron stores [32] whereas sTfR is a measure of functional iron availability to body tissues, including the brain. Therefore, sTfR may give a more accurate “read-out” of brain iron availability. Using a model of the blood-brain barrier, it has been shown that iron transport into the brain is not associated with ferritin concentration in peripheral blood [33]. Further, under certain conditions functional iron deficiency can occur even if iron stores are adequate, due to complex regulatory mechanisms [34]. The release of iron from stores into circulation, is regulated by hepcidin, not only in response to circulating iron availability, but also other factors including inflammation [34]. Iron stimulates the growth of most human pathogens, impacting susceptibility and severity of infections [35]. Therefore, under inflammatory conditions, hepcidin prevents the release of iron stores from hepatocytes and macrophages and prevents absorption of iron in the gut, limiting the supply of iron to the invading pathogen [34]. Even very low-grade inflammation, which is known to be widespread within this population of The Gambia [36], is associated with hepcidin stimulation. Therefore, although this physiological response is protective from infectious disease, it can reduce availability of iron for essential functions such as the processes of neurodevelopment, even if iron stores are adequate. Although sTfR can also be impacted by inflammatory status [37], studies suggest that it provides a more robust indication of iron demand under inflammatory conditions than does ferritin [38,39].

This is not the first study to report the relationship between cognitive measures and sTfR; The MAL-ED study [40], also recently reported that lower mean sTfR over the first two years of life was associated with better performance in a standardised cognitive assessment at five years of age (Bayley Scales of Infant Development), whereas ferritin and haemoglobin were not [41]. Although, the MAL-ED analyses were based on mean sTfR concentration in blood samples taken at 7, 15 and 24 months of age, and therefore do not reflect early infancy specifically, this finding supports the results presented within the current study, and provides evidence that the impact of iron deficiency early in life extends to pre-school age.

In previous studies, behavioural assessments have been shown to lack sensitivity to the impacts of developmental risk factors when used under 2 years of age [42]. However, in this study, when implemented at 5 months of age, the MSEL was sensitive to developmental differences associated with early iron status. Visual disengagement time was also sensitive to attentional differences between terciles of sTfR and allowed at least partial insight into the mechanisms by which iron may impact the developing brain. Our findings suggest that iron status in early infancy may directly impact the development of attentional processing, the foundation of learning, memory and social interaction [43,44], which then has broader consequences for cognitive development more generally. This mediating role of attention in the relationship between iron status and cognition was also identified in a recent study by East et al. [45] who reported that iron deficiency in infancy (6–18 months of age) was associated with inattention at 10 years of age and poorer educational achievement at 21 years of age within a longitudinal cohort in Chile. A clearer insight into the mechanisms by which iron deficiency impacts the developing brain will help inform intervention design.

This study has several strengths. Firstly, it represents one of the most comprehensive longitudinal assessments of the relationship between iron status and neurodevelopment across the first 1000 days of life, a critical period for brain development [46]. Further, it focuses specifically on early iron status (under 6 months of age), which is currently underrepresented within the literature [47]. In addition, this study was conducted in sub-Saharan Africa where the burden of infant iron deficiency is highest, yet previous research in this field is sparse. Finally, these analyses utilised a combination of methods to assess neurodevelopment; a behavioural assessment of global cognition and an objective measure of attention which is mechanistically linked to iron [48].

However, some limitations must also be noted. Firstly, despite robust statistical approaches and inclusion of a wide range of potential confounding factors, this analysis is based on observational data which are prone to bias and does not, therefore, show causality. Further, assessment of other micronutrients was not included within the models presented. Micronutrient deficiencies often co-occur, and many other micronutrients including iodine, zinc and copper have roles in neurodevelopmental processes. The relationship between iron status and neurodevelopment within this study may, therefore, be attributed to other nutritional deficiencies, or other unmeasured confounding effects [46,49,50]. Although the relatively small sample size is also a limitation, this study represents one of the largest longitudinal cohorts where neurodevelopment has been assessed at multiple timepoints, including very early in infancy. The participants in the BRIGHT study were recruited from a rural Gambian community, which relied largely on subsistence farming and among which parental education and socioeconomic status were mostly very low. Therefore, the findings from this study may not be generalisable to populations with characteristics very different from this one. However, infants and young children in sub-Saharan Africa are among the most at risk of iron deficiency and are currently under-represented in the literature [11], so despite limitations in generalisability, this study makes a valuable contribution to the field.

Finally, although iron status is an important factor in neurocognitive development, and iron status in early infancy is a previously neglected topic which warrants increased focus, it is important to acknowledge that the factors influencing neurocognitive development are many and wide ranging. The windows of opportunity to support early child development and mitigate the impacts of early adverse experiences extend beyond ensuring iron sufficiency in the first 6 months of life.

In conclusion, this study provides evidence that functional iron status (meased by sTfR) within early infancy (0–6 months of age) is associated with neurodevelopmental outcomes across infancy and early childhood, within a population in sub-Saharan Africa where iron deficiency is common. We also identified one possible mechanism (attentional processing) by which this relationship is established. Interventions to maintain adequate iron and supply of other essential micronutrients throughout pregnancy and early infancy, alongside other aspects of nurturing care, are likely to yield neurodevelopmental benefits, with long lasting impact.

## Supporting information

S1 Fig Histogram of residuals from final model of MSEL cognitive score. (DOCX)

S2 Fig Q normal plot of residuals in final MSEL cognitive score model. (DOCX)

S3 Fig Scatter plot of residuals from MSEL cognitive score model with age. (DOCX)

S4 Fig Histogram of residuals from final model of visual disengagement time. (DOCX)

S5 Fig Q normal plot of residuals from disengagement time model with age. (DOCX)

S6 Fig Scatter plot of residuals from visual disengagement time model with age. (DOCX)

S7 Fig Prevalence of iron deficiency by exclusive breastfeeding status at 5 months of age. (DOCX)

S1 Table Collection of analysis of blood samples. (DOCX)

S2 Table Percentage of attending infants with valid outcome data from 5mo to 3–5 yrs. (DOCX)

S3 Table Model of MSEL cognitive score trajectories including terciles of 5mo Hb. (DOCX)

S4 Table Model of MSEL cognitive score trajectories including terciles of 5mo sTfR. (DOCX)

S5 Table Model of MSEL cognitive score trajectories including terciles of 5mo log ferritin. (DOCX)

S6 Table Model of visual disengagement time trajectories including terciles of 5mo Hb. (DOCX)

S7 Table Model of visual disengagement time trajectories including terciles of 5mo sTfR. (DOCX)

S8 Table Model of visual disengagement time trajectories including terciles of 5mo ferritin. (DOCX)

S1 Text Supplementary methods. (DOCX)
