# Supplementary material for: Iron status in early infancy is associated with trajectories of cognitive development up to pre-school age in rural Gambia
Source: PLOS Glob Public Health. 2023 Nov 1;3(11):e0002531. doi: 10.1371/journal.pgph.0002531 (PMC10619872; doi:10.1371/journal.pgph.0002531)
Supplement: S1 Fig — (DOCX) [file pgph.0002531.s001.docx]

**Figure S1 Histogram of Residuals from final model of MSEL Cognitive Score**
