# Supplementary material for: Iron status in early infancy is associated with trajectories of cognitive development up to pre-school age in rural Gambia
Source: PLOS Glob Public Health. 2023 Nov 1;3(11):e0002531. doi: 10.1371/journal.pgph.0002531 (PMC10619872; doi:10.1371/journal.pgph.0002531)
Supplement: S7 Fig — (DOCX) [file pgph.0002531.s007.docx]

Figure S7 Prevalence of Iron Deficiency by Exclusive Breastfeeding Status at 5 months of age

EB= Exclusive breastfeeding

P=0.051
