# Supplementary material for: Iron status in early infancy is associated with trajectories of cognitive development up to pre-school age in rural Gambia
Source: PLOS Glob Public Health. 2023 Nov 1;3(11):e0002531. doi: 10.1371/journal.pgph.0002531 (PMC10619872; doi:10.1371/journal.pgph.0002531)
Supplement: S1 Table — (DOCX) [file pgph.0002531.s008.docx]

**Table S1 Collection of Analysis of Blood Samples**

|  | **1M**  **N (% attended)** | **5M**  **N (% attended)** | **8M**  **N (% attended)** | **12M**  **N (% attended)** |
| --- | --- | --- | --- | --- |
| No. Infants Attended | 185 | 195 | 188 | 188 |
| Blood sample collected | 183 (98.9) | 192 (98.5) | 178 (94.7) | 184 (97.9) |
| Haemoglobin | 170 (91.9) | 181 (92.8) | 172 (91.5) | 173 (92.0) |
| Complete iron/ inflammatory markers. | 126 (68.1) | 179 (91.8) | 161 (85.6) | 154 (81.9) |

The Table shows the number and percentage of infants with blood samples and iron/ inflammatory markers from 1-12mo. CRP; c-reactive protein.
