# Supplementary material for: Iron status in early infancy is associated with trajectories of cognitive development up to pre-school age in rural Gambia
Source: PLOS Glob Public Health. 2023 Nov 1;3(11):e0002531. doi: 10.1371/journal.pgph.0002531 (PMC10619872; doi:10.1371/journal.pgph.0002531)
Supplement: S2 Table — (DOCX) [file pgph.0002531.s009.docx]

**Table S2 Percentage of Attending Infants with Valid Outcome Data from 5mo to 3-5 yrs**

|  | **Attended**  **(No. infants)** | **MSEL Cognitive Score**  **% of attended** | **Disengagement Time**  **% of attended** |
| --- | --- | --- | --- |
| 5 months of age | \| **195** \| \| --- \| | 80.5 | 76.9 |
| 8 months of age | **188** | 87.2 | 80.3 |
| 12 months of age | **187** | 85.6 | 71.7 |
| 18 months of age | **177** | 85.9 | 75.7 |
| 24 months of age | **164** | 86.0 | 64.6 |
| 3-5 years of age | **171** | 84.2 | 97.7 |

The table shows the percentage of attending infants with valid data for MSEL cognitive score and eye-tracking disengagement time at each time point, as well as the percentage of infants who completed all cognitive subscales and for whom the ELC score could be calculated.
