# Supplementary material for: Iron status in early infancy is associated with trajectories of cognitive development up to pre-school age in rural Gambia
Source: PLOS Glob Public Health. 2023 Nov 1;3(11):e0002531. doi: 10.1371/journal.pgph.0002531 (PMC10619872; doi:10.1371/journal.pgph.0002531)
Supplement: S3 Table — (DOCX) [file pgph.0002531.s010.docx]

**Table S3 Model of MSEL Cognitive Score Trajectories Including Terciles of 5mo Hb**

| MSEL Cognitive Score | **Co-eff** | **Std. Error** | **P>\|z\|** | **95% CI** | |
| --- | --- | --- | --- | --- | --- |
| ***Observations= 837***  ***Infants = 177***  ***Mean observation per infant= 4.7*** |  |  |  | Lower Bounds | Upper Bounds |
| Age | 2.94 | 0.05 | **<0.001** | 2.83 | 3.04 |
| Age^3^ | -0.01 | 0.00 | **<0.001** | -0.01 | 0.00 |
| 5mo Hb Medium | -0.92 | 0.65 | 0.157 | -2.21 | 0.36 |
| 5mo Hb High | 0.48 | 0.68 | 0.481 | -0.85 | 1.81 |
| Age_Hb Medium | 0.15 | 0.70 | **0.034** | 0.01 | 0.28 |
| Age_Hb High | 0.10 | 0.07 | 0.145 | -0.36 | 0.24 |
| Log CRP (5mo) | -0.08 | 0.14 | 0.570 | -0.36 | 0.20 |
| Constant | 27.05 | 0.49 | **<0.001** | 26.10 | 28.01 |
| *Random Effects* |  |  |  |  |  |
| Variance (Age) | 0.11 | 0.01 | - | 0.09 | 0.15 |
| Variance (constant) | 2.92 | 1.46 | - | 1.10 | 7.80 |
| Covariance (Age, Constant) | -0.39 | 0.12 | - | -0.62 | -0.16 |
| Variance (Residual) | 23.17 | 1.48 | - | 20.45 | 26.26 |
