# Supplementary material for: Iron status in early infancy is associated with trajectories of cognitive development up to pre-school age in rural Gambia
Source: PLOS Glob Public Health. 2023 Nov 1;3(11):e0002531. doi: 10.1371/journal.pgph.0002531 (PMC10619872; doi:10.1371/journal.pgph.0002531)
Supplement: S4 Table — (DOCX) [file pgph.0002531.s011.docx]

**Table S4 Model of MSEL Cognitive Score Trajectories Including Terciles of 5mo sTfR**

| MSEL Cognitive Score | **Co-eff** | **Std. Error** | **P>\|z\|** | **95% CI** | |
| --- | --- | --- | --- | --- | --- |
| ***Observations= 837***  ***Infants = 177***  ***Mean observation per infant= 4.7*** |  |  |  | Lower Bound | Upper Bound |
| Age | 3.05 | 0.06 | **<0.001** | 2.94 | -3.16 |
| Age^3^ | -0.01 | 0.00 | **<0.001** | -0.01 | 0.00 |
| 5mo sTfR Low | 2.00 | 0.66 | **0.003** | 0.70 | 3.30 |
| 5mo sTfR Medium | 1.39 | 0.64 | **0.031** | 0.13 | 2.65 |
| Age_ sTfR Low | -0.03 | 0.07 | 0.658 | -0.18 | 0.11 |
| Age_ sTfR Medium | -0.09 | 0.07 | 0.198 | -0.23 | 0.05 |
| Log CRP (5mo) | -0.13 | 0.13 | 0.333 | -0.40 | 0.14 |
| Constant | 25.82 | 0.48 | **<0.001** | 24.88 | 26.77 |
| *Random Effects* |  |  |  |  |  |
| Variance (Age) | 0.12 | 0.02 | - | 0.09 | -0.15 |
| Variance (constant) | 2.78 | 1.42 | - | 1.02 | 7.58 |
| Covariance (Age, Constant) | -0.40 | 0.11 | - | -0.63 | -0.18 |
| Variance (Residual) | 23.05 | 1.46 | - | 20.35 | 26.10 |
