# Supplementary material for: Iron status in early infancy is associated with trajectories of cognitive development up to pre-school age in rural Gambia
Source: PLOS Glob Public Health. 2023 Nov 1;3(11):e0002531. doi: 10.1371/journal.pgph.0002531 (PMC10619872; doi:10.1371/journal.pgph.0002531)
Supplement: S5 Table — (DOCX) [file pgph.0002531.s012.docx]

**Table S5 Model of MSEL Cognitive Score Trajectories Including Terciles of 5mo Log Ferritin**

| MSEL Cognitive Score | **Co-eff** | **Std. Error** | **P>\|z\|** | **95% CI** | |
| --- | --- | --- | --- | --- | --- |
| ***Observations= 837***  ***Infants = 177***  ***Mean observation per infant= 4.7*** |  |  |  | Lower Bound | Upper Bound |
| **Fixed Effects** |  |  |  |  |  |
| Age | 3.04 | 0.06 | **<0.001** | 2.93 | 3.15 |
| Age^3^ | -0.01 | 0.00 | **<0.001** | -0.01 | 0.00 |
| 5mo log Ferritin Medium | 0.08 | 0.68 | 0.902 | -1.24 | 1.41 |
| 5mo log Ferritin High | 0.25 | 0.67 | 0.709 | -1.05 | 1.55 |
| Age_ log Ferritin Medium | -0.05 | 0.07 | 0.532 | -0.19 | 0.10 |
| Age_ log Ferritin High | -0.02 | 0.07 | 0.793 | -0.16 | 0.12 |
| Log CRP (5mo) | -0.11 | 0.14 | 0.420 | -0.39 | 0.16 |
| Constant | 26.79 | 0.50 | **<0.001** | 25.81 | 27.77 |
| *Random Effects* |  |  |  |  |  |
| Variance (Age) | 0.12 | 0.02 | - | 0.09 | 0.15 |
| Variance (constant) | 3.36 | 1.50 | - | 1.40 | 8.05 |
| Covariance (Age, Constant) | -0.41 | 0.12 | - | -0.65 | -0.18 |
| Variance (Residual) | 23.10 | 1.47 | - | 20.39 | 26.16 |
