# Supplementary material for: Iron status in early infancy is associated with trajectories of cognitive development up to pre-school age in rural Gambia
Source: PLOS Glob Public Health. 2023 Nov 1;3(11):e0002531. doi: 10.1371/journal.pgph.0002531 (PMC10619872; doi:10.1371/journal.pgph.0002531)
Supplement: S6 Table — (DOCX) [file pgph.0002531.s013.docx]

**Table S6 Model of Visual Disengagement Time Trajectories Including Terciles of 5mo Hb**

| Disengagement | Co-eff | Std. Error | P>\|z\| | 95% CI | |
| --- | --- | --- | --- | --- | --- |
| **Obs=721**  **Infants= 179**  **Avg Obs/ Infant= 4.0** |  |  |  | Lower Bound | Upper Bound |
| Age | 0.49 | 0.55 | 0.368 | -0.58 | 1.56 |
| Ln(Age) | -18.00 | 2.25 | **<0.001** | -22.42 | -13.58 |
| (Ln Age)^2^ | -5.23 | 1.39 | **<0.001** | -7.96 | -2.51 |
| 5mo Hb Medium | -6.24 | 12.96 | 0.630 | -31.65 | 19.17 |
| 5mo Hb High | -32.93 | 13.43 | **0.014** | -59.24 | -6.61 |
| Age_ Hb Medium | 0.21 | 0.37 | 0.566 | -0.51 | 0.94 |
| Age_ Hb High | 0.81 | 0.39 | **0.037** | 0.05 | 1.57 |
| Log CRP (5mo) | 2.48 | 2.28 | 0.276 | -1.99 | 6.96 |
| Constant | 156.62 | 9.71 | **<0.001** | 137.59 | 175.65 |
| *Random Effects* |  |  |  |  |  |
| Variance (Age) | 0.91 | 0.33 |  | 0.45 | 1.86 |
| Variance (Constant) | 3253.30 | 561.73 |  | 2319.27 | 4563.48 |
| Covariance | -54.39 | 13.65 |  | -81.13 | -27.65 |
| Variance Residual | 3677.64 | 224.14 |  | 3263.56 | 4144.26 |
