# Supplementary material for: Iron status in early infancy is associated with trajectories of cognitive development up to pre-school age in rural Gambia
Source: PLOS Glob Public Health. 2023 Nov 1;3(11):e0002531. doi: 10.1371/journal.pgph.0002531 (PMC10619872; doi:10.1371/journal.pgph.0002531)
Supplement: S7 Table — (DOCX) [file pgph.0002531.s014.docx]

**Table S7 Model of Visual Disengagement Time Trajectories Including Terciles of 5mo sTfR**

| Disengagement | Co-eff | Std. Error | P>\|z\| | 95% CI | |
| --- | --- | --- | --- | --- | --- |
| **Obs= 490**  **Infants= 126**  **Avg Obs/ Infant= 3.9** |  |  |  | Lower Bound | Upper Bound |
| Age | 0.45 | 0.55 | 0.415 | -0.63 | 1.52 |
| Ln(Age) | -18.19 | 2.25 | **<0.001** | -22.60 | -13.77 |
| (Ln Age)^2^ | -5.41 | 1.39 | **<0.001** | -8.13 | -2.69 |
| 5 mo sTfR Low | -46.53 | 13.15 | **<0.001** | -72.31 | -20.74 |
| 5 mo sTfR Medium | -26.03 | 13.14 | **0.048** | -51.78 | -0.28 |
| Age_ sTfR Low | 0.86 | 0.39 | **0.028** | 0.09 | 1.62 |
| Age_ sTfR Medium | 0.35 | 0.38 | 0.357 | -0.40 | 1.10 |
| Log CRP (5mo) | 2.87 | 2.23 | 0.199 | -1.51 | 7.25 |
| Constant | 170.07 | 10.35 | **<0.001** | 149.78 | 190.37 |
| *Random Effects* |  |  |  |  |  |
| Variance (Age) | 0.93 | 0.34 | - | 0.45 | 1.90 |
| Variance (Constant) | 3120.71 | 548.16 | - | 2221.29 | 4412.47 |
| Covariance | -53.85 | 13.61 | - | -80.52 | -27.19 |
| Variance Residual | 3674.37 | 223.85 | - | 3260.83 | 4140.37 |
