# Supplementary material for: Iron status in early infancy is associated with trajectories of cognitive development up to pre-school age in rural Gambia
Source: PLOS Glob Public Health. 2023 Nov 1;3(11):e0002531. doi: 10.1371/journal.pgph.0002531 (PMC10619872; doi:10.1371/journal.pgph.0002531)
Supplement: S8 Table — (DOCX) [file pgph.0002531.s015.docx]

**Table S8 Model of Visual Disengagement Time Trajectories Including Terciles of 5mo Ferritin**

| Disengagement | Co-eff | Std. Error | P>\|z\| | 95% CI | |
| --- | --- | --- | --- | --- | --- |
| ***Obs=721***  ***Infants= 179***  ***Avg Obs/ Infant= 4.0*** |  |  |  | Lower Bound | Upper Bound |
| Age | 0.70 | 0.55 | 0.200 | -0.37 | 1.77 |
| Ln(Age) | -18.19 | 2.26 | **<0.001** | -22.62 | -13.77 |
| (Ln Age)^2^ | -5.32 | 1.39 | **<0.001** | -8.05 | -2.59 |
| 5mo Ferritin Medium | -13.99 | 13.41 | 0.297 | -40.27 | 12.29 |
| 5mo Ferritin High | -17.09 | 13.44 | 0.204 | -43.43 | 9.26 |
| Age_ Ferritin Medium | 0.11 | 0.39 | 0.770 | -0.64 | 0.87 |
| Age_ Ferritin High | 0.30 | 0.39 | 0.432 | -0.45 | 1.06 |
| Log CRP (5mo) | 3.31 | 2.24 | 0.140 | -1.08 | 7.70 |
| Constant | 155.33 | 10.27 | **<0.001** | 135.20 | 175.45 |
| ***Random Effects*** |  |  |  |  |  |
| Variance (Age) | 1.03 | 0.36 |  | 0.52 | 2.03 |
| Variance (Constant) | 3431.94 | 579.63 |  | 2464.79 | 4778.60 |
| Covariance | -59.48 | 14.26 |  | -87.43 | -31.53 |
| Variance Residual | 3674.69 | 223.79 |  | 3261.24 | 4140.56 |
