# Supplementary material for: Iron status in early infancy is associated with trajectories of cognitive development up to pre-school age in rural Gambia
Source: PLOS Glob Public Health. 2023 Nov 1;3(11):e0002531. doi: 10.1371/journal.pgph.0002531 (PMC10619872; doi:10.1371/journal.pgph.0002531)
Supplement: S1 Text — (DOCX) [file pgph.0002531.s016.docx]

***Eye tracking Disengagement Time.***

Eye tracking is a non-invasive technique with broad applications in research into visual attention. The eye tracker used within this study was the Tobii TX300 (Tobii Technology Stockholm, Sweden), with 300 Hz refresh rate and integrated 23-inch monitor was used to record eye movement. This system is an optical tracking system comprising (i) a source of near infrared light, which illuminates the eyes, causing reflections of the cornea and pupil, (ii) a camera to capture an image of these reflections and (iii) algorithms to calculate gaze direction based on geometric features of the corneal and pupil reflections. Visual stimuli were presented to the infant on the integrated screen and audio was played through loud speakers. The eye tracker communicated with a Macbook pro (OS X 10.9) running MATLAB 8.0.

Within a screen-based assessment, timestamps of gaze direction and duration were overlaid on the presented stimuli to determine how eye movements related to the presented stimuli [1].

***Stimulus and Assessment Procedure***

Before the assessment started, the infant/ child was positioned on their mother’s lap around 70cm from the screen. The eye-tracker was calibrated to the infant’s eye movements using a rotating spiral stimulus, which was presented in each of the four corners and centre of the screen in turn, accompanied by an engaging sound. The infant’s position was adjusted if necessary to ensure the eye tracker picked up their eye movements accurately. Following successful calibration, the infant watched the stimuli on the screen for the duration of the assessment. If they became distracted, an audio attention grabber was played to redirect their attention to the screen. If they became uninterested or fussy, the assessment was paused, and the infant/ took a short break before restarting if possible. Throughout the assessment the infant/child was observed via web cam.

The gap-overlap paradigm was based on a task developed by Elsabbagh et al. [2, 3] and was one component of the larger eye tracking battery within the BRIGHT study. Gap overlap trials started by presenting a pulsating stimulus (a cartoon clock) in the centre of the screen to attract the infant’s attention. When gaze fixation on the stimulus was detected, the stimulus started to spin to maintain their attention. After 600-700ms, a peripheral stimulus was presented to the left or right side of the screen in one of three conditions: (i) baseline, (i) gap, and (iii) overlap. In the baseline condition, the peripheral stimulus appeared, and central stimulus disappeared simultaneously, in the gap condition the central stimulus disappeared 200ms prior to the appearance of the peripheral stimulus, reducing the competition for attention. In the overlap condition, the central stimulus remained on the screen, but stopped rotating, when the peripheral stimulus appeared, increasing the competition for attention. The peripheral stimulus remained on the screen until gaze fixation was detected, or until 2000ms had passed at which point it became animated and engaging sounds (e.g. car horn or bell) were played for 1000ms. Fifteen trials in each condition were presented and only infants/ children with at least four valid trials in each condition were included in analysis.

*Data Preparation and Analysis*

Data were cleaned using an automated process to remove gaze co-ordinates that corresponded to a distance greater than one screen away and data with a validity code over 1, indicating uncertainty regarding which eye the co-ordinates were reporting, were removed. Fixations were then identified using the noise-robust identification by 2-means clustering algorithm [4].

Mean saccadic reaction time, that is the time between the onset of the peripheral stimulus and fixation on that stimulus, was calculated for each infant for two of the conditions (baseline and overlap) at each time point. These reaction times were then used to calculate the disengagement component: Disengagement was defined as the difference in saccadic reaction time between the baseline and the overlap conditions (overlap – baseline) and represented the infant’s ability to switch between competing stimuli.

***Variables included in the mixed effects models***

Infant sex: Studies have shown that boys are more at risk of iron deficiency than girls [5], and are more likely to underachieve in developmental assessments [6].

Season of assessment: Iron status among infants in The Gambia varies by season [7], as does maternal nutritional status in pregnancy which may lead to differential developmental outcomes, depending on the time of year that an infant is born and subsequently assessed [8].

Maternal education: There is good evidence that parental education and associated household income is related to neurodevelopmental trajectories in terms of both brain structure and function [9] and children of more highly educated parents have previously found to be at lower risk of anaemia and iron deficiency [10].

Infant length: There is a strong relationship between childhood stunting and cognitive development [6] and the inverse relationship between iron status and linear growth, in which rapid growth is associated with poorer iron status [11, 12].

Gestational age at birth: Infants born at lower gestational age at birth are at higher risk of receiving inadequate iron endowment in utero and therefore becoming iron deficient in infancy [13] and gestational age is also independently associated with developmental outcomes, even among babies born at term [14].

CRP: CRP was included in the model because increased inflammation impacts iron homeostasis and has also been linked to alterations in neurodevelopmental processes [15]. Widespread chronic low-grade inflammation has previously been reported among infants and young children in The Gambia [16].

**References**

1. Jian-nan, C., et al. *Key Techniques of Eye Gaze Tracking Based on Pupil Corneal Reflection*. in *2009 WRI Global Congress on Intelligent Systems*. 2009.

2. Elsabbagh, M., et al., *Disengagement of visual attention in infancy is associated with emerging autism in toddlerhood.* Biol Psychiatry, 2013. **74**(3): p. 189-94.

3. Elsabbagh, M., et al., *Visual orienting in the early broader autism phenotype: disengagement and facilitation.* J Child Psychol Psychiatry, 2009. **50**(5): p. 637-42.

4. Hessels, R.S., et al., *Noise-robust fixation detection in eye movement data: Identification by two-means clustering (I2MC).* Behavior Research Methods, 2017. **49**(5): p. 1802-1823.

5. Antunes, H., et al., *Male gender is an important clinical risk factor for iron deficiency in healthy infants.* e-SPEN Journal, 2012. **7**(6): p. e219-e222.

6. McCoy, D.C., et al., *Early Childhood Developmental Status in Low- and Middle-Income Countries: National, Regional, and Global Prevalence Estimates Using Predictive Modeling.* PLoS Med, 2016. **13**(6): p. e1002034.

7. Atkinson, S.H., et al., *Combinatorial effects of malaria season, iron deficiency, and inflammation determine plasma hepcidin concentration in African children.* Blood, 2014. **123**(21): p. 3221-3229.

8. Moore, S.E., *Early life nutritional programming of health and disease in The Gambia.* Journal of developmental origins of health and disease, 2016. **7**(2): p. 123-131.

9. Noble, K.G., et al., *Family income, parental education and brain structure in children and adolescents.* Nature Neuroscience, 2015. **18**(5): p. 773-778.

10. Choi, H.-J., et al., *Effects of maternal education on diet, anemia, and iron deficiency in Korean school-aged children.* BMC public health, 2011. **11**: p. 870-870.

11. Perng, W., et al., *Iron status and linear growth: a prospective study in school-age children.* Eur J Clin Nutr, 2013. **67**(6): p. 646-51.

12. McCarthy, E.K., et al., *Iron intakes and status of 2-year-old children in the Cork BASELINE Birth Cohort Study.* Maternal and Child Nutrition, 2017. **13**(3).

13. Moreno-Fernandez, J., et al., *Iron Deficiency and Iron Homeostasis in Low Birth Weight Preterm Infants: A Systematic Review.* Nutrients, 2019. **11**(5): p. 16.

14. Hua, J., et al., *Differentiating the cognitive development of early-term births in infants and toddlers: a cross-sectional study in China.* BMJ Open, 2019. **9**(4): p. e025275.

15. Adelantado-Renau, M., M.R. Beltran-Valls, and D. Moliner-Urdiales, *Inflammation and Cognition in Children and Adolescents: A Call for Action.* Frontiers in pediatrics, 2020. **8**: p. 583-583.

16. Prentice, A.M., et al., *Respiratory infections drive hepcidin-mediated blockade of iron absorption leading to iron deficiency anemia in African children.* Science Advances, 2019. **5**(3): p. eaav9020.
